# *In vitro* and *In vivo* Model Systems for Hemophilia A Gene Therapy

**DOI:** 10.4172/2157-7412.S1-014

**Published:** 2013-01-17

**Authors:** Jianhua Mao, Xiaodong Xi, Philipp Kapranov, Biao Dong, Jenni Firrman, Ruian Xu, Weidong Xiao

**Affiliations:** 1 Shanghai Institute of Hematology, Rui Jin Hospital, Shanghai Jiao Tong University School of Medicine, Shanghai 200025, China; 2 St. Laurent Institute, One Kendall Square, Cambridge, MA, USA; 3 Department of Microbiology and Immunology, Sol Sherry Thrombosis Research Center, Temple University, Philadelphia, PA, USA; 4 Institute of Molecular Medicine, Molecular Medicine Engineering Research Center, Huaqiao University, Quanzhou 362021, China

**Keywords:** Human gene therapy, Factor VIII, Hereditary disorder, Hemophilia A sheep

## Abstract

Hemophilia A is a hereditary disorder caused by various mutations in factor VIII gene resulting in either a severe deficit or total lack of the corresponding activity. Recent success in gene therapy of a related disease, hemophilia B, gives new hope that similar success can be achieved for hemophilia A as well. To develop a gene therapy strategy for the latter, a variety of model systems are needed to evaluate molecular engineering of the factor VIII gene, vector delivery efficacy and safety-related issues. Typically, a tissue culture cell line is the most convenient way to get a preliminary glimpse of the potential of a vector delivery strategy. It is then followed by extensive testing in hemophilia A mouse and dog models. Newly developed hemophilia A sheep may provide yet another tool for evaluation of factor VIII gene delivery vectors. Hemophilia models based on other species may also be developed since hemophiliac animals have been identified or generated in rat, pig, cattle and horse. Although a genetic nonhuman primate hemophilia A model has yet to be developed, the non-genetic hemophilia A model can also be used for special purposes when specific questions need to be addressed that cannot not be answered in other model systems. Hemophilia A is caused by a functional deficiency in the factor VIII gene. This X-linked, recessive bleeding disorder affects approximately 1 in 5000 males [1–3]. Clinically, it is characterized by frequent and spontaneous joint hemorrhages, easy bruising and prolonged bleeding time. The coagulation activity of FVIII dictates severity of the clinical symptoms. Approximately 50% of all cases are classified as severe with less than 1% of normal levels of factor VIII detected [4]. This deficiency may lead to spontaneous joint hemorrhages or life-threatening bleeding. In contrast, patients with 5–30% of normal factor VIII activity exhibit mild clinical manifestations.

## Introduction

Protein replacement therapy is currently the mainstay for effective treatment of hemophilia A [5,6]. The administration of recombinant factor VIII avoids many of the complications associated with plasma derived factor VIII protein, including the risk of infectious diseases. Nevertheless, frequent infusions of recombinant protein are not only expensive but also come with a risk of inhibitor formation, which occurs in 15% to 30% of hemophilia A patient receiving replacement therapy [7]. Human gene therapy remains an ideal cure which could avoid all these complications and improve the quality of life for hemophilia A patients. Recent successes in gene therapy of hemophilia B, Leber congenital amaurosis (LCA), lipoprotein lipase deficiency and severe combined immunodeficiency suggest that hemophilia A gene therapy may be achieved as well [8–13].

The F8 gene which encodes the factor VIII polypeptide has been located in the X-chromosome at Xq28, spans approximately 180 Kb and includes 26 exons. Analysis of the F8 gene sequences from hemophilia A patients demonstrated that a large variety of mutations throughout the gene can lead to a severe hemophilia phenotype [14]. Approximately half of severe hemophilia A patients have intron 22 inversion, which is most likely caused by a homologous, intra-chromosomal recombination event between a 9. 6 kb sequence within the F8 gene intron 22 and one of the two inversely orientated homologous sequences located 300–500 kb distal to the F8 gene [15–17]. All other types of mutations that are known to cause hemophilia A do not exhibit the same dominant genetic hot spot in the factor VIII coding regions. Mutations in the introns, or even the non-essential B-domain regions can also lead to a hemophilia phenotype [14].

As with any other human disease, it is essential to have model systems that faithfully recapitulate the effects of deficiency in factor VIII for both hemophilia A basic research as well as for development of novel treatments. A recent review by Sabatino et al. extensively covered the common available mouse and dog models [18]. Here we will offer a complementary discussion of additional model systems available for hemophilia A research.

## *In vitro* Tissue Culture Systems

Systems based on cultured cells provide the most economical way of testing factor VIII function as well as allow for preliminary vector characterizations for the gene therapy strategies. The translated FVIII polypeptide contains 2,351 amino acids that has six functional domains A1-A2-B-A3-C1-C2 in addition to the 19 amino acids signal peptide [19,20]. Full-length factor VIII is a secreted glycoprotein of 280 kDa that typically undergoes extensive post-translational processing and modifications. Shorter versions of factor VIII containing only A1-A2-A3-C1-C2 domains (B-domain deleted factor VIII) are commonly used for gene therapy due to improved RNA synthesis and stability [21]. However, performance of these shorter versions of factor VIII in vector-based gene delivery applications were not satisfactory using either lentiviral vectors or AAV vectors. Extensive factor VIII engineering is believed to be necessary to achieve maximal delivery efficiency [22]. Throughout this process, the gene delivery constructs containing factor VIII variants can be conveniently characterized in the *in vitro* cultured cells.

Although factor VIII is predominately synthesized and secreted by the hepatocytes or sinusoidal endothelial cells in the liver, most *in vitro* characterization or production has utilized non-human cell lines including baby hamster kidney cells (BHK), Chinese hamster ovary (CHO) cell line, BHK or COS cells. The CHO cell line is the primary cell line used for producing commercial factor VIII products Advate (Baxter. Advate^®^ prescribing Information. Deerfield: Baxter, 2010), Xyntha [23] and Refacto (Wyeth Pharma (Pfizer). ReFacto^®^ Prescribing Information. Madison: Wyeth Pharma (Pfizer), 2007). BHK cells have been used for producing Kogenate [24]. On the other hand, Cos-7 cells have been mainly used in basic research related to the factor VIII protein biology. All these cell lines are believed to allow for proper factor VIII post-translational modifications and thus ensure adequate factor VIII coagulation activities.

One major factor that dictates selection of specific lines of cells for testing factor VIII expression is their ability to properly secrete the factor VIII polypeptide peptide. Human HEK 293 cells had been thought to poorly support factor VIII expression. However, new studies have suggested that this is not the case, especially for the factor VIII with deleted B-domain. The factor VIII produced in human 293 cells may have additional advantages over cells of non-human origin in terms of post-translational modification [25,26]. In these studies, it was noted that factor VIII expressed from 293 cells was sulfated and glycosylated comparable to the human plasma-derived factor VIII. Furthermore, the antigenic Neu5Gc or alpha-Gal epitopes observed in CHO- and BHK-derived rFVIII products were absent from factor VIII produced by the human HEK 293 cells. Thus, it would be especially advantageous to initially test a gene therapy strategy in a cell line of human origin.

Induced pluripotent stem cells (iPS) are *in vitro* cultured cells that may be used for *ex vivo* gene therapy. Expression of transcription factors such as Oct-4, Sox2, Klf4 and Myc can turn somatic cells, such as the fibroblast, into the iPS cells by using ES medium containing several cytokines [27–33]. The resulting iPS cells can be induced and differentiated into hepatocytes, endothelial cells or endothelial progenitor cells, which may express the endogenous factor VIII gene directly. Subsequently, the endothelial progenitor cells derived from iPS cells can be engrafted into the liver of a hemophilia mouse model and achieve phenotypic correction of the bleeding disorder [34]. Although factor VIII expression *in vitro* is not the objective in the stem cell culture system *per se*, factor VIII expression can be achieved *in vivo* upon differentiation. In this case, iPS cells become a vehicle for factor VIII gene [35]. After the iPS-derived cells were injected directly into the liver of irradiated hemophilia A mice, plasma FVIII levels increased to 8% to 12% of the wild type level in transplanted hemophilia A mice and corrected the hemophilia A phenotype. Because the iPS cells may be originated from the patient’s own somatic cells it could greatly reduce immune rejection, which gives them a tremendous therapeutic benefit.

Besides iPS cells, the use of hematopoietic stem cells (HSC) has also been explored as an *ex vivo* strategy for factor VIII expression using integrating vectors [36,37]. Transplanting modified HSCs expressing B domain deleted porcine FVIII (BDD-pFVIII) into hemophilia A mice corrected the bleeding disorder [36,38–41]. However, successful HSC transplation-based gene therapy for hemophilia A hinges on the development of low radiation-based intensity conditioning regimens to control toxicity. A potential for secondary malignant transformation due to insertional mutagenesis caused by retroviral gene transfer is another concern. The development of insulated, self-inactivating gamma retroviral vectors with an enhancer-blocking element can potentially decrease genotoxicity of retroviral integration [42].

Similar to iPS cells and hematopoietic stem cells, genetically modified mesenchymal stem cells (MSC) are a promising target for delivery of secreted protein due to their ease of isolation, expansion and genetic modification. Based on these advantages, MSC have been tested in several human clinical trials with some reported success. For example, allogeneic MSC cells were used in a clinical trial to treatment a genetic collagen disorder osteogenesis imperfecta [43–45]. In addition to treating diseases of mesenchymal origin, the suitability of MSC as a cellular vehicle for gene-transfer applications has also been studied [46,47]. MSC transduced with FVIII-encoding retroviral or lentiviral vectors were able to achieve a high level expression of FVIII and reduce the bleeding phenotype of hemophilia A mice after transplantation [48,49]. *In vivo*, MSC do not get transformed or progress to clonal dominance following transduction with integrating viral vectors. Rather, these cells undergo terminal differentiation rather than transformation in the presence of DNA damage [50]. This very attractive property separates MSC from the hematopoietic cells or iPS cells in gene therapy applications.

## Hemophilia A Rodent Models

The commonly used factor VIII knockout strains of mice were created by inserting a *neo* expression cassette in exon 16 (E16−/− line) and 17 (E17−/− line) of the factor VIII gene. Molecular characterization of these mice revealed no detectable level of factor VIII. However, the strains exhibited a much milder phenotype than that of the factor IX knockout mouse (hemophilia B). The E16−/− mice may not exhibit frequent spontaneous bleeding which contrasts to what has been observed in hemophilia A patients. The bleeding phenotype in this mouse model is primarily observed after tail clipping, which resulted in roughly 70% fatality in the affected male mice. This model has been extensively used in hemophilia A related studies and has been recently reviewed [18].

Additional mouse models related to this line have been produced, one particular model is the von Willebrand factor (vWF)-fVIII double knockout strain (vWF^null^FVIII^null^) [51,52]. vWF is a multimeric glycoprotein that serves as a carrier protein for factor VIII. It binds to factor VIII in multiple regions including the acidic region 3 (a3) and light chain thus forming a non-covalently linked complex. vWF stabilizes factor VIII by inhibiting phospholipid-binding proteins that target factor VIII for proteolytic degradation, prevents cellular uptake of factor VIII via scavenger cell receptors and extends factor VIII half-life in circulation. The half-life of FVIII is reduced to less than three hours in patients with the type 3 von Willebrand diseases. The protective effect of vWF on factor VIII was also manifested by the fact that anti-FVIII antibodies resulting from infusion of factor VIII concentrates into the hemophilia A mouse model that has either totally lacks or has a lower level of vWF predominantly recognize the acidic a1 and a3 regions where vWF would have normally been bound [53]. Since hemophilia A patients would not be deficient in vWF, the vWF^null^FVIII^null^ strain can be used in a special situation to evaluate the factor VIII pharmacokinetics in the absence of vWF, which is especially valuable when engineered factor VIII may have altered interaction with vWF.

Another albeit less common line of factor VIII knock-out mice was generated by deleting factor VIII exons 16–19 (E16–19 (−/−)) [54]. The initial molecular characterization confirmed that the desired genetic modifications were introduced at the DNA level. The resulting factor VIII mRNA from E16–19 (−/−) demonstrated the anticipated 750 bp deletion. Surprisingly, it was reported that the FVIII: C activities in heterozygous, hemizygous and homozygous mice were at 80%, 8% and 10% of the level in normal mice respectively. We analyzed the factor VIII activity in this line by the chromogenic assay and determined that the homozygous mice actually retained only 0.7% of the wild-type FVIII activity. Thus, it indeed exhibited a true hemophilia phenotype and may be valuable for gene transfer experiments, especially for researchers in China where the other hemophilia A mouse model is not available. The discrepancy between there ported result based on one stage a PTT and our chromogenic assay is likely due to the same discrepancy in one- and two-stage activity assays observed in the rat model [55]. Therefore, it may be inferred that some defective factor VIII was synthesized in the E16–19(−/) strain being the cause of this discrepancy. The disagreement between the results of 1-stagevs2-stage activity assays of mutated human factor VIII has also been reported by Pipe et al. [56]. There is also a possibility that the immune response toward the vector or the factor VIII itself in this strain may be different from the E16(−/) strain. However, more analyses will be necessary to show the reason for these differences.

Hemophiliac WAG-F8^m1Ycb^ rats have also been reported [55,57]. Periarticular hemorrhage, spontaneous bruising, prolonged bleeding from minor wounds and maternal peripartum deaths are common in the affected male and female rats. These symptoms are much more severe than what has been observed in the hemophiliac mice. Genetically, the hemophiliac phenotype in these rats was determined to be caused by the Leu176Pro substitution in the rat factor VIII gene. This mutation was thought to affect the functionality of factor VIII protein rather than its secretion. Overall, hemophiliac rats will be an interesting model for gene delivery related studies if related reagents can be developed [58].

## Fully-Characterized Large Animal Hemophilia A Models

While the hemophilia A mouse models have been most convenient and useful for initial studies, there is a consensus that they did not fully and accurately recapitulate the human disease. Many aspects of gene transfer outcomes (i.e: vector dose, immune responses to the transgene, immune responses to vectors, etc.) in small animal models differ from what would be encountered in human patients. It is therefore necessary to move gene transfer studies into a larger animal model to obtain testing results which are more statistically and physiologically meaningful.

The hemophilia A dog is the most characterized large hemophilia A model. There are currently two well-maintained colonies with one maintained at UNC Chapel Hill and the other at Queens University [59–63]. Defective factor VIII activity in both colonies arises from the intron 22 inversion, which accounts for nearly 50% of severe human hemophilia A cases. Preclinical testing results of factor VIII related treatments in this model have been documented to be excellent indicators of later clinical efficacy in humans. In similar hemophilia B dog model, results of gene therapy approaches are largely consistent with the outcome of human clinical trials. The dog model has been used for many studies related to adenoviral, AAV, and retroviral delivery of FVIII. Readers are encouraged to refer to the extensive coverage of this model by Sabatino et al. [18].

Beside the commonly used hemophilia A dog model, another large animal model is the newly developed hemophiliac sheep [64,65]. Hemophiliac sheep were first reported in the male offspring of a flock of White Alpine sheep at ETH-Swiss Federal Institute of Technology, Zürich. Their genetic defect in factor VIII gene was confirmed by restriction fragment length polymorphism (RFLP) analysis [66–69]. The hemophilia A sheep line was re-established using the preserved semen obtained before the animals’ demise [64,65]. Molecular characterization revealed that an 11 bp region in exon 14 differed between the wild-type and the hemophiliac animals. This alteration introduced a premature stop codon at base position 3112–4 in exon 14. In addition, it contained a single nucleotide insertion which caused a frame shift and multiple following stop codons.

Phenotypically, the recreated hemophiliac sheep exhibited prolonged bleeding from the umbilical cord at the birth. Severe internal bleeding caused by birth trauma requires factor VIII infusion treatment. Such treatment led to the formation of inhibitor against human factor VIII. Bleeding after tail clipping, ear tagging or after trimming hooves was also observed as well as spontaneous hemarthrosis, muscle hematomas and hematuria that also occurred frequently in these animals. All these clinical symptoms resembled those caused by the human disease. The FVIII:C activity of the affected HA sheep is about 2.3%, which was the limit of the sensitivity of the aPTT test used to quantitate the levels of FVIII:C by the authors. The actual FVIII:C activity therefore could be lower than this reported number as suggested by the chromogenic assay which showed no active FVIII in circulation of that sheep. Recombinant ovine factor VIII has been developed to preserve and maintain the sheep HA colony since the sheep developed immune responses to human FVIII proteins thus rendering the protein treatments ineffective [70]. Recombinant ovine factor VIII with B domain deletion produced from baby hamster cells exhibited therapeutic effectiveness in the hemophilia A mouse model. However, even if treatment with the recombinant ovine factor VIII is effective and avoids the formation of inhibitors in the affected sheep, similar to the efficiency of treatment with canine factor VIII to maintain the hemophiliac dogs, it remains a challenge since the cost of treating large sheep (75 kg) with ovine FVIII might be similar to the cost of treating humans. However, this sheep colony is still the only fully characterized model in which frequent recurrent hemarthroses are common and thus represents a valuable model of this clinically important aspect of the human disease. It is therefore important that more resources are made available and more gene delivery studies carried out in this model. Another study attempted to reverse the sheep hemophilia A phenotype with the postnatal intraperitoneal transplantation of FVIII-expressing MSC. Haploidentical (paternal) bone marrow (BM)-derived MSC were engineered to express porcine FVIII using a lentiviral vector. The two hemophilia A sheep receiving this treatment exhibited a significant improvement in existent hemarthroses and damaged joints while spontaneous bleeds ceased. Despite phenotypic correction, no FVIII activity was detected in the circulation of the two treated animals presumably because MSC migrated to sites of injury and released FVIII locally to correct existing hemarthroses. Nevertheless, a high titer of factor VIII inhibitors developed in these two animals and nullified the effectiveness of the treatment. Further investigations are needed to identify the cause of the inhibitor formation in order to avoid similar problems during human gene therapy clinical trials.

Porcine model of hemophilia A generated by nuclear transfer cloning was recently reported [71]. The resulting hemophilia A pig contained similar mutations as the E16(−/−) factor VIII knock-out mice caused by the neomycin-resistance gene (PGK-neo) insertion into exon 16. Molecular characterization confirmed that canonical factor VIII mRNA was absent in the liver of piglets with the knockout genotype. Phenotypically, large hematomas and massive traumatic intramuscular bleeding were observed in the affected piglets. Human factor VIII infusion in the piglets led to inhibitors formation. So far, the characterization of this animal model appeared to be rather preliminary. However, since porcine factor VIII have been studied extensively [72], it might be relatively easier to use porcine hemophilia A model for gene therapy related studies.

As discussed above, there appears to be a choice of large animal models for hemophilia A related gene therapy studies. Interspecies differences will have to be evaluated carefully before the selection. Factors to be considered in the choice of the model system include body size, physiology, disease progression and manifestation, genetic background such as the nature of factor VIII mutations and sequence homology to humans. Besides these scientific factors, economic considerations and availability of these animals often dominate choice of model to be used for a gene delivery study.

## Other Potential Hemophilia A Models

For the sake of completeness, we wish to review yet another group of animal models that have potential for use as hemophilia A gene therapy models, but were, however, not sufficiently developed and lost for various reasons. The first case of a hemophiliac cow was reported in Herefords [73], but the causative mutation has not yet been identified. A more thoroughly studied case was reported in Japan [74], where a female, hemophilia A Japanese Brown cattle dam carrier was identified and two hemophiliac offspring calves were obtained. One cow had no obvious bleeding and eventually suffered from a hematoma at 7 months. The unclotted blood in the hematomas took up to 60 minutes to form a clot in a plain plastic tube. The other cattle developed swelling containing unclotted blood between the lower jaw and the chest at 4 months and died within three days after the onset of the swelling. The genetic characterization of hemophilia A cattle was also reported [75]. The causative mutation was narrowed down to one nucleotide substitution of T to A at position 6459 in mRNA (6459T→A), changing Leucine at position 2153 in the protein to Histidine (p. Leu2153His). This is a highly conserved amino acid residue in the C1 domain. Since there was no spontaneous bleeding such as hemarthrosis and ecchymoma in the cattle, they were not considered as severe types of hemophilia [73].

A hemophilia A cat has also been reported [76], however there is no molecular characterization of its genotype. In addition, since “a recommendation was made that the dam be tested and/or be neutered as soon as possible”, it is most likely that this hemophiliac line has been lost and thus it is unlikely to make a contribution to gene therapy. Based on the initial report, the cat had no history suggestive of spontaneous bleeding which indicates that the factor VIII deficiency in the cat most likely resembled that of the mouse. Similar to the hemophilia A cat case, hemophilia A horses were briefly reported on multiple occasions [77–79].

Although hemophilia A may be found in any species, and valuable information can be obtained from these cases, developing these sporadic cases into an animal model useful for gene therapy testing is not easily accomplished. After reviewing the model development history of the hemophilia A mouse and dog, we have to agree that significant research investment is required to develop species-specific reagents, assays and experience, all of which would be required before results generated in a newer model could be received and evaluated properly [58].

## Non Genetic Model for Hemophilia Research

Logically, it is desirable to use an animal model with a factor VIII genetic defect for hemophilia gene therapy since such animals may mimic human disease and the effects of gene delivery can be determined without the interference of endogenously expressed factor VIII protein. However, a non-genetic model without an inheritable factor VIII deficiency may be used under special circumstances. Transient hemophilic rabbit models can be created by infusing plasma containing FVIII inhibitors [80]. This can be very valuable for testing and developing FVIII bypass reagents, which are important therapeutics for hemophilia A patients with inhibitors [81–83].

Although no factor VIII deficient non-human primate (NHP) has been discovered, they can still be used in gene therapy experiments for testing the effectiveness of gene transfer vectors. There are two types of testing that can be done on non-human primates, even though they have no defects in the factor VIII gene *per se*. First, vector potency and toxicity can be determined. Similar studies have been carried out with factor IX gene delivery [84–89]. Second, transgene expression, i.e., factor VIII levels produced by the vector can be measured. In one reported study an adenovirus carrying an epitope-tagged human factor VIII cDNA was administered and the expression of factor VIII in the plasma of four male cynomologus monkeys receiving the vector was determined through immunoprecipitation and Western blotting [90]. Even though in this instance, the experimental animal still expresses its own endogenous factor VIII, expression from administered vectors can be determined and other aspects related to gene delivery can be studied using the animal model closest to humans. It is also worth noting that the important immunological aspects of factor VIII gene delivery, i.e., inhibitor formation to recombinant protein or vector-derived FVIII, cannot be adequately addressed in NHP with the endogenous wild type factor VIII gene.

In summary, there are many models systems available for testing factor VIII delivery technology. While the *in vitro* tissue culture cells remain the most convenient system for initial testing, the information obtained is rather limited. It is a standard routine that factor VIII vectors are to be tested in the mouse models before moving into the more expensive and well characterized hemophilia A dog model. The hemophilia A sheep is an interesting model that remains to be validated and explored. Non-human primate for hemophilia A would resemble most human disease and it would probably be best predicator of factor VIII vector performance in humans. Until a true hemophilia A non-primate can be identified, creative ways are needed to deplete monkey factor VIII so that the unmodified/native human factor VIII expression can be measured accurately.
